# Oncotype DX Recurrence Score Predicts Survival in Invasive Micropapillary Breast Carcinoma: A National Cancer Database Analysis

**DOI:** 10.3390/curroncol32100559

**Published:** 2025-10-05

**Authors:** Ali J. Haider, Mohummad Kazmi, Kyle Chang, Waqar M. Haque, Efstathia Polychronopoulou, Jonathon S. Cummock, Sandra S. Hatch, Andrew M. Farach, Upendra Parvathaneni, E. Brian Butler, Bin S. Teh

**Affiliations:** 1 Department of Radiation Oncology, University of Texas Medical Branch, Galveston, TX 77555, USA; alhaider@utmb.edu (A.J.H.); shatch@utmb.edu (S.S.H.); upparvat@utmb.edu (U.P.); 2 Texas College of Osteopathic Medicine, University of North Texas Health Science Center, Fort Worth, TX 76107, USA; mohummadkazmi@my.unthsc.edu; 3 John Sealy School of Medicine, University of Texas Medical Branch, Galveston, TX 77555, USA; khchang@utmb.edu; 4 Department of Radiation Oncology, Houston Methodist Hospital, Houston, TX 77030, USA; wmhaque@houstonmethodist.org (W.M.H.); amfarach@houstonmethodist.org (A.M.F.); ebutler@houstonmethodist.org (E.B.B.); 5 Department of Biostatistics & Data Science, University of Texas Medical Branch, Galveston, TX 77555, USA; efpolych@utmb.edu; 6 Naresh K. Vashisht College of Medicine, Texas A&M University, Bryan, TX 77807, USA; jcummock@houstonmethodist.org

**Keywords:** oncotype DX, invasive micropapillary carcinoma, breast cancer, recurrence score, prognosis, radiation therapy, survival

## Abstract

**Simple Summary:**

Invasive micropapillary carcinoma (IMPC) is a rare and aggressive form of breast cancer that often spreads to lymph nodes early on. The 21-gene Oncotype DX test estimates risk of recurrence and guides chemotherapy use in common hormone-receptor-positive breast cancers, but its value in IMPC is unknown. Using the U.S. National Cancer Database, we identified women with early-stage IMPC who received Oncotype DX testing and were not treated before surgery. Patients with high scores (>25) had worse five-year survival than those with low (≤11) or intermediate (12–25) scores, while low and intermediate groups had similarly excellent outcomes. The assay therefore helps identify IMPC patients at higher risk and may inform decisions about adjuvant therapy, including radiation.

**Abstract:**

(1) Background: Invasive micropapillary carcinoma (IMPC) is a rare, aggressive breast cancer subtype marked by high lymph node metastasis rates. While Oncotype DX recurrence score (RS) offers prognostic information for patients with hormone-receptor-positive (HR+) breast cancer, its utility in IMPC—a histology with distinct biologic behavior—remains unvalidated. This study evaluates whether Oncotype DX offers prognostic information with respect to overall survival (OS) in non-metastatic, early-stage patients with IMPC of the breast. (2) Methods: The National Cancer Database (2004–2020) was queried to select for women with ER+/HER2−, T1-T2N0-N1 IMPC who underwent Oncotype DX testing and received no neoadjuvant therapy. Patients were stratified by RS: low (≤11), intermediate (12–25), and high (>25). Kaplan–Meier survival curves and log-rank tests compared 5-year OS between groups. Multivariable Cox proportional hazards models assessed RS as an independent predictor, adjusting for age, race, comorbidities, grade, radiation, and insurance status. (3) Results: A total of 1325 women met the selection criteria. The cohort demonstrated significant survival disparities by RS (log-rank *p* = 0.017). Five-year OS rates were 97.5%, 97.5%, and 93.7% for low, intermediate, and high-risk patients, respectively. Adjusted multivariate analysis confirmed RS as an independent prognosticator: low (HR = 0.31, 95% CI: 0.15–0.75) and intermediate (HR = 0.32, 95% CI: 0.15–0.75) scores correlated with reduced mortality versus high RS. Omission of radiation therapy (HR = 2.68, 95% CI: 1.05–6.86) and higher comorbidity burden (0 comorbidities vs. ≥2: HR = 0.25, 95% CI: 0.10–0.61) were significantly associated with worse survival. (4) Conclusions: Oncotype DX is predictive for OS in IMPC, with high RS (>25) portending poorer outcomes. The survival detriment associated with RT omission aligns with prior studies demonstrating RT benefit in higher-risk cohorts. These findings validate RS as a prognostic tool in IMPC and underscore its potential to refine adjuvant therapy, particularly RT utilization. Future studies should explore RS-driven treatment personalization in IMPC, including comorbidity management and adjuvant radiation to improve outcomes in this distinct patient population.

## 1. Introduction

Invasive micropapillary carcinoma (IMPC) of the breast is a rare, histologically aggressive subtype of breast cancer notable for frequent lymphovascular invasion and lymph node involvement, even in small, early-stage tumors [1,2,3]. Despite its distinct biological behavior, IMPC is often managed similarly to invasive ductal carcinoma (IDC) of no special type, guided by clinicopathological factors such as hormone receptor status, tumor grade, and proliferation indices [4,5]. However, emerging evidence suggests that IMPC exhibits unique molecular features and clinical outcomes, including elevated rates of locoregional and distant recurrence, underscoring the need for more individualized prognostic assessment [6,7].

The 21-gene Recurrence Score (Oncotype DX) assay has transformed risk stratification in hormone-receptor-positive (HR+), HER2-negative early-stage breast cancer, predicting distant recurrence risk and chemotherapy benefit [8,9,10]. Trials including TAILORx and RxPONDER demonstrated that patients with low genomic risk (RS < 25) derived minimal chemotherapy benefit, while identifying significant chemotherapy benefit primarily for patients with high-risk scores (RS ≥ 26) [10,11,12]. For node-positive disease, the SWOG S8814 trial demonstrated that RS predicts chemotherapy benefit independently of nodal burden, with high RS patients showing significant improvements in disease-free survival, while those with low RS derived limited to no advantage [13]. These landmark studies validated the integration of tumor biology into clinical management guidelines for IDC [14,15].

Despite the well-established utility of RS in IDC, its predictive value for biologically distinct subtypes such as IMPC remains unproven. IMPC’s aggressive phenotype, marked by paradoxical clinical behavior where even small tumors exhibit high nodal involvement, raises questions about whether genomic assays validated in IDC are generalizable to this population [6,16]. Additionally, the role of RS in guiding adjuvant radiation therapy (RT) remains unexplored in IMPC, despite evidence from IDC populations suggesting differential RT benefits according to RS [17,18,19]. Beyond Oncotype DX, other multi-gene assays such as MammaPrint (70-gene signature), Prosigna (PAM50), and EndoPredict (12-gene score) also provide prognostic and predictive information for HR+ breast cancer. However, their utility specifically in IMPC remains largely unproven, underscoring the need to validate genomic risk stratification in this unique biologic context.

Previous analyses of the National Cancer Database (NCDB) by our group identified RS as an indicator of potential radiation benefit among older patients (≥70 years) and node-negative cohorts with IDC [20,21]. However, these analyses did not stratify rare subtypes like IMPC, leaving a gap in understanding how genomic assays perform in biologically distinct malignancies. Given IMPC’s propensity for early metastasis and recurrence [3], clarifying the prognostic value of RS in this population is essential to refine risk-adapted therapy, including RT intensification or de-escalation.

In this study, we evaluate the predictive utility of the Oncotype DX recurrence score in a large, contemporary cohort of non-metastatic IMPC patients using the NCDB. We hypothesize that high RS will correlate with inferior survival outcomes, independent of traditional clinicopathological factors, and that RT omission will disproportionately impact high-risk subgroups. By validating the prognostic capability of Oncotype DX in this unique population, our findings aim to inform personalized adjuvant strategies and address an unmet clinical need in the management of IMPC.

## 2. Materials and Methods

Data Source and Study Populations: Data for this retrospective cohort study were extracted from the National Cancer Database (NCDB; 2004–2020), a collaborative initiative between the American College of Surgeons’ Commission on Cancer and the American Cancer Society. The NCDB aggregates de-identified records from more than 1500 accredited U.S. cancer programs, encompassing approximately 70% of all new cancer diagnoses nationwide. These data include detailed information on patient demographics, tumor characteristics, and survival outcomes, though rural and minority populations are underrepresented due to the database’s non-population-based design. All analyzed data were obtained from a participant user file with no personal identifiers, rendering the study exempt from institutional review board oversight [22]. The datasets supporting this study’s conclusions are accessible from the corresponding author upon request.

**Figure 1:**
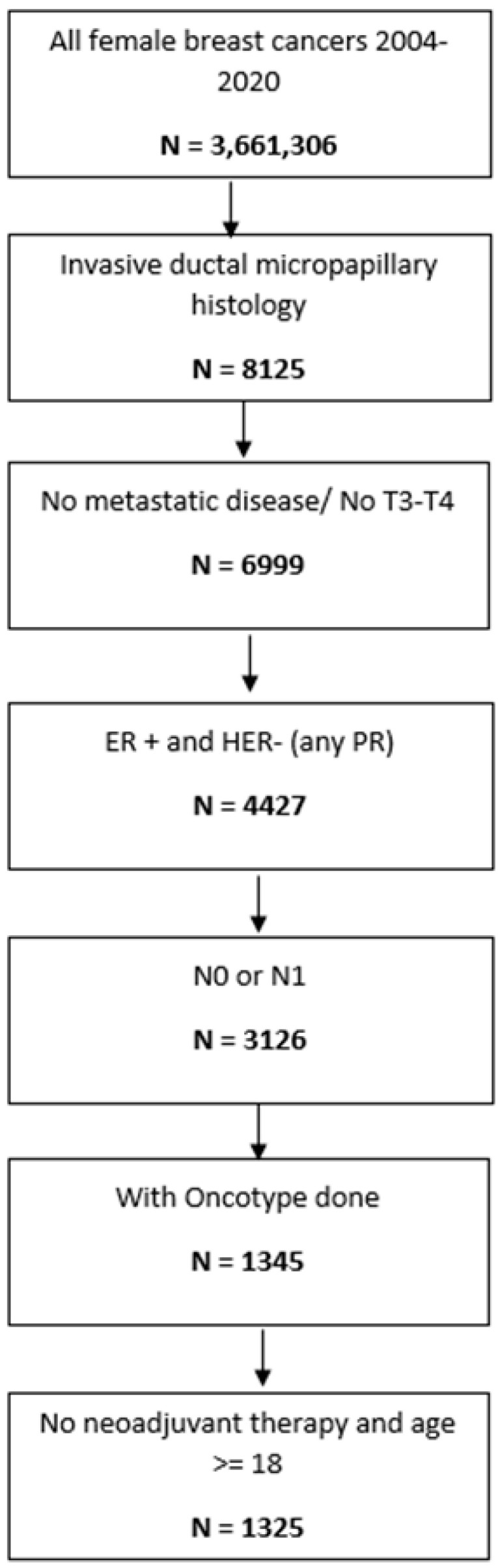
Patient selection flowchart for ER+/HER2− invasive micropapillary carcinoma (IMPC; N = 1325) from the National Cancer Database (2004–2020). Exclusion applied for metastatic disease, neoadjuvant therapy, and missing Oncotype DX data.

Patient Selection and Cohorts: Using the NCDB (2004–2020), 8125 patients were identified having IMPC from 3,661,306 female patients having breast cancer. After excluding metastatic (M1) and locally advanced (T3–T4) disease, 6999 patients remained. Further refinement included hormone-receptor-positive (ER+), HER2-negative tumors, with nodal status N0–N1 and available Oncotype DX recurrence scores, resulting in 1345 patients. Figure selection is demonstrated in Figure 1. Patients receiving neoadjuvant therapy were excluded, yielding a final population of 1325 women aged ≥18 years old with non-metastatic IMPC treated with upfront surgery. Patients were then stratified into three groups by recurrence score categories: low (≤11), intermediate (12–25), and high (>25). Baseline clinicopathologic characteristics of the study cohort are summarized in Table 1.

**Figure 2:**
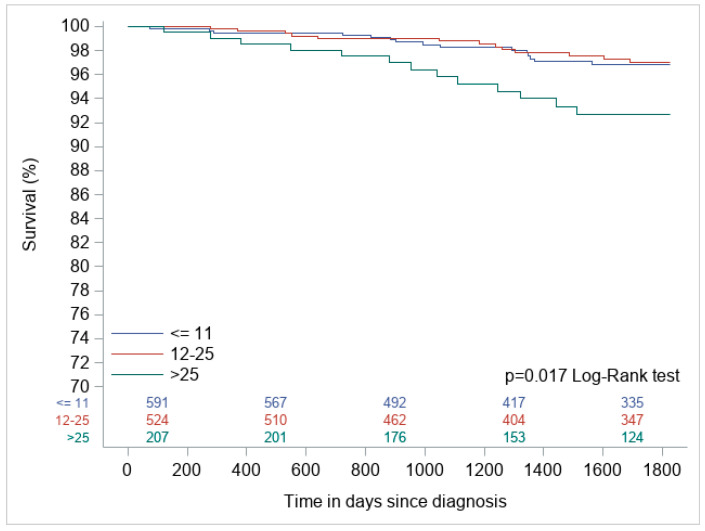
Kaplan–Meier overall survival analysis by Oncotype DX recurrence score (RS) groups in invasive micropapillary carcinoma (log-rank *p* = 0.017). High RS (>25) showed significantly worse 5-year survival (93.7%) versus low (≤11) and intermediate (12–25) RS groups (both 97.5%).

Statistical Procedures: The primary endpoint was 5-year overall survival (OS). OS was defined as the time from diagnosis to death from any cause. Subjects were censored at loss of follow-up or 5 years from diagnosis, whichever occurred first. Survival distributions for the three groups were compared using Kaplan–Meier survival analysis (shown in Figure 2), with statistical significance assessed via the log-rank test.

To evaluate the independent association between Oncotype DX recurrence scores and OS while adjusting for potential confounders, a multivariable Cox proportional hazards model was employed. The model incorporated age, comorbidities, race, grade, insurance, surgery type, and radiation therapy status as covariates. Age was dichotomized at 60 years as a standard surrogate for menopausal status in registry analyses. The effect of adjuvant chemotherapy on OS, as well as the interaction between chemotherapy and RS group, was also evaluated. Results are presented as hazard ratios with corresponding 95% confidence intervals, with a *p*-value threshold of 0.05 for statistical significance.

## 3. Results

From the National Cancer Database (2004–2020), 1325 women with ER+/HER2−, T1-T2N0-N1 invasive micropapillary carcinoma (IMPC) who underwent Oncotype DX testing and received no neoadjuvant therapy were analyzed. The cohort was stratified by recurrence score (RS): low (≤11; n = 593, 44.8%), intermediate (12–25; n = 525, 39.6%), and high (>25; n = 207, 15.6%). Baseline demographics were balanced across RS groups, with no significant differences in age, race, comorbidity burden, or treatment modality (Table 2). Chemotherapy utilization varied across RS groups but was not significantly associated with overall survival (*p* = 0.82).

Five-year overall survival (OS) differed significantly by RS (log-rank *p* = 0.017). Patients with low (97.5%) and intermediate (97.5%) RS had nearly identical survival rates, while high RS (>25) was associated with inferior 5-year OS (93.7%) and a 6.2% 5-year mortality rate—nearly triple that of low/intermediate groups (2.4–2.5%) (Table 2, Figure 2).

On adjusted Cox regression, low (HR = 0.31, 95% CI: 0.15–0.75) and intermediate RS (HR = 0.32, 95% CI: 0.15–0.75) were independently associated with reduced mortality risk compared to high RS (reference) (Table 3). Chemotherapy administration was not significantly associated with survival benefit (HR = 1.12, 95% CI: 0.41–3.00; *p* = 0.82), and an interaction test between Oncotype group and chemotherapy yielded no significance (*p* = 0.3). Omission of radiation therapy (HR = 2.68, 95% CI: 1.05–6.86; *p* = 0.039) and higher comorbidity burden (0 comorbidities vs. ≥2: HR = 0.25, 95% CI: 0.10–0.61; *p* = 0.002) were the other significant predictors of worse survival. Age, race, tumor grade, insurance status, and surgical approach (lumpectomy vs. mastectomy) did not significantly impact survival (all *p* > 0.05).

**Figure 3:**
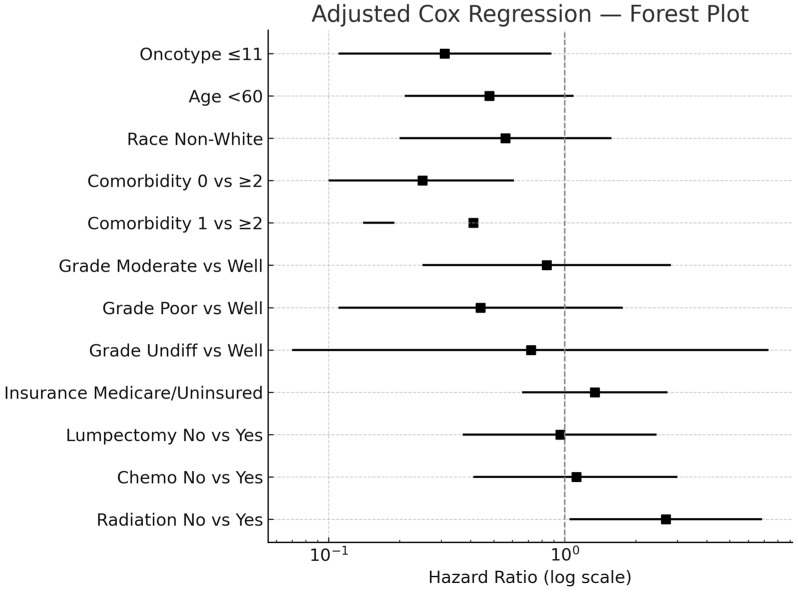
Forest plot of multivariable Cox regression analysis for overall survival in patients with ER+/HER2− IMPC treated with upfront surgery and Oncotype DX testing. Hazard ratios (HR) with 95% confidence intervals (CI) are shown on a log scale for recurrence score group, age, race, comorbidities, grade, insurance, surgery type, chemotherapy, and radiation.

Table 3 shows the results of multivariable Cox regression analysis of 5-year survival in invasive micropapillary carcinoma (N = 1325). Low (HR = 0.31) and intermediate (HR = 0.32) Oncotype DX scores were associated with reduced mortality versus high scores (>25). These findings are visually represented in Figure 3. Radiation omission (HR = 2.68) and comorbidities (≥2 vs. 0: HR = 0.25) were significant predictors.

The survival detriment associated with high RS (>25) persisted across nodal status (N0 vs. N1) and age groups (<60 vs. ≥60 years). Notably, the lack of OS difference between low and intermediate RS groups contrasted with prior studies in invasive ductal carcinoma, suggesting potential limitations of conventional RS thresholds in IMPC.

## 4. Discussion

Our analysis provides evidence supporting the predictive ability of the Oncotype DX 21-gene RS assay within IMPC. Specifically, we found that IMPC patients with high genomic risk (RS > 25) experienced significantly poorer overall survival, validating the relevance of Oncotype DX in this distinct histology. In contrast, patients with low (≤11) and intermediate (12–25) RS had nearly identical 5-year survival (~97.5% in both groups). High-RS tumors (>25) were associated with almost triple the 5-year mortality of low/intermediate-RS tumors (6.2% vs. ~2.4%). Critically, multivariable analysis demonstrated that chemotherapy administration did not significantly improve survival (HR = 1.12, 95% CI: 0.41–3.00; *p* >> 0.05), nor was there evidence of differential benefit by RS group (interaction *p* = 0.3).

Notably, our multivariate analysis revealed that adjuvant chemotherapy did not significantly improve survival in this IMPC cohort (HR = 1.12, *p* = 0.82), nor was there evidence of differential chemotherapy benefit by Oncotype group (interaction *p* = 0.3). This contrasts with prior studies in IDC, where high RS tumors derive clear chemotherapy benefit [10,11]. The lack of association may reflect IMPC’s unique biology, which could render it less responsive to conventional chemotherapy regimens, or potential could reflect confounding by unmeasured variables (e.g., treatment adherence, dose reductions). Alternatively, the relatively small high-RS subgroup (n = 207) may have limited power to detect a modest survival difference. Further research is needed to clarify whether IMPC-specific therapeutic approaches, such as novel agents targeting its micropapillary architecture, might offer greater efficacy.

This suggests that the survival disadvantage observed in high-RS patients reflects inherent tumor aggressiveness rather than differential treatment effects. These results indicate that while a high RS clearly portends worse outcomes in IMPC, mirroring its established adverse significance in conventional breast cancer, the distinction between low and intermediate RS categories may not translate into a meaningful survival difference in this aggressive subtype.

The prognostic value of the 21-gene assay across rare histologic subtypes, which often exhibit distinct biological behaviors, is an area of ongoing investigation. Our findings in IMPC align with a large-scale genomic analysis by Tadros et al., which evaluated Recurrence Score distributions among traditional “favorable-prognosis” subtypes, including tubular, mucinous, papillary, and cribriform carcinomas [23]. Within these subtypes, the proportion of patients with a high-risk RS (using a cutoff of >25) ranged from 3.2% in tubular carcinoma to over 18% in papillary carcinoma, suggesting intrinsic biologic diversity. In this setting, histologic classification and genomic profiling can potentially serve as complementary risk stratification tools. Extending this principle to IMPC, we demonstrate that within this aggressive phenotype, the Recurrence Score effectively identifies a subset of patients with high-risk biology and significantly inferior survival, reinforcing the assay’s utility beyond common ductal carcinomas.

Several factors may explain the lack of prognostic separation between low and intermediate RS IMPC. One possibility is that the current RS cutoffs, originally derived from more common, less aggressive histology, are not optimally calibrated for IMPC’s biology. Oncotype DX was developed and validated primarily in patients with IDC and lobular carcinoma, and our findings suggest its prognostic “granularity” may be reduced in IMPC. Biologically, IMPC’s inherent aggressiveness (e.g., frequent lymphovascular invasion and nodal metastasis) could diminish the incremental risk conferred by an intermediate RS; essentially, even genomic “low-risk” IMPC might have a baseline elevated risk of relapse. It is also plausible that treatment patterns influenced our observations; clinicians may have been inclined to administer adjuvant chemotherapy to IMPC patients with intermediate scores out of concern for the histology, thereby improving their outcomes to mirror those of the low-RS group. This possibility is supported by the higher chemotherapy utilization observed in the current dataset in the intermediate group compared to the low-risk group. However, in our analysis, interaction testing did not demonstrate a significant OS benefit from chemotherapy in this cohort. This phenomenon would be consistent with our finding that low- and intermediate-RS IMPC had indistinguishable survival. Together, these considerations raise the question of whether IMPC might benefit from modified RS thresholds. For example, a lower cutoff to define “high risk” (or conversely, treating intermediate scores as high-risk in IMPC) could be explored to better stratify patients, though such an approach requires prospective validation.

Our findings can be contextualized alongside the landmark studies that established the clinical utility of the 21-gene assay in the general HR+/HER2− breast cancer population. The seminal work by Paik et al. (2004) first validated the prognostic impact of the Recurrence Score (RS) in tamoxifen-treated, node-negative breast cancer, demonstrating that the assay could stratify distant recurrence risk independent of clinical factors [8]. The NSABP B-20 trial first demonstrated the predictive value of the RS for chemotherapy benefit in node-negative, ER-positive breast cancer; patients with high RS (using the original cutoff ≥31) derived a large absolute benefit from adjuvant chemotherapy, whereas those with low RS (<18) gained virtually no benefit. The subsequent TAILORx trial refined these risk categories and prospectively confirmed that endocrine therapy alone is noninferior to chemoendocrine therapy for patients with mid-range scores [10]. In TAILORx, women with RS 11–25 had outcomes with hormonal therapy alone that were as good as those with added chemotherapy (9-year freedom from distant recurrence ~95% in both arms), leading to the recommendation that most postmenopausal patients in this intermediate genomic risk group can safely forgo chemotherapy [24]. Our observation that IMPC patients with RS ≤ 25 achieve excellent 5-year survival aligns with the TAILORx findings; it suggests that, despite IMPC’s aggressive histology, cases deemed low or intermediate risk by Oncotype have favorable prognoses, at least in the short term. It must be noted, however, that neither B-20 nor TAILORx specifically evaluated special histologic subtypes like IMPC. The overwhelming majority of tumors in those trials were IDC-NST, and thus the applicability of their conclusions to IMPC has remained somewhat uncertain. Our study helps bridge this gap by showing that the 21-gene assay’s risk stratification has relevance in IMPC (particularly at the high end of the score spectrum), but it also underscores that IMPC may not exhibit the same gradations of risk across the low–intermediate range as seen in conventional tumors.

Notably, the role of Oncotype DX in node-positive disease has been clarified by recent evidence, which is pertinent given that over half of IMPC patients present with nodal metastases [25]. The SWOG-8814 and RxPONDER trials extended the 21-gene assay’s applicability to patients with 1–3 positive lymph nodes [11,13]. In these studies, the RS was prognostic and predictive even in the presence of limited nodal disease, and results suggest that the majority of postmenopausal patients with 1–3 positive nodes and RS ≤ 25 can be spared chemotherapy without compromising outcomes [11]. On the other hand, premenopausal patients with node-positive disease and intermediate scores appeared to derive a modest benefit from chemotherapy [11]. Our NCDB cohort included both node-negative and node-positive (N1) IMPC, and the RS remained an independent predictor of survival in multivariable analysis regardless of nodal status. This consistency with prior node-positive studies implies that the biological significance of a high RS in driving outcomes holds true in IMPC, even when regional spread has occurred.

The limitations of our retrospective analysis highlight avenues for future research. Firstly, our use of overall survival (OS) as the endpoint (in the absence of cancer-specific survival data in NCDB) may underestimate the RS’s ability to predict breast cancer recurrence or mortality. Prior SEER-based analyses, for instance, reported no significant breast cancer–specific survival differences by RS in IMPC, possibly due to limited sample size or short follow-up for this rare subtype [23]. Although our study demonstrates a clear OS separation for high RS, confirming the prognostic value of Oncotype in IMPC, it remains unknown whether different RS thresholds would improve the assay’s discriminatory power for this subtype. Prospective validation is needed to determine if, for example, an IMPC patient with an RS of 20 truly has the same indolent course as one with an RS of 5 (as our data suggest), and whether any intermediate-RS IMPC tumors might benefit from escalation of therapy despite falling below the traditional high-risk cutoff. Future studies should consider incorporating IMPC cases into dedicated clinical trials or registries, to test if adjusted RS cut points could better stratify risk. In addition, deeper molecular characterization of IMPC may reveal genomic alterations or expression patterns not captured by the 21-gene panel, information that could be leveraged to design more tailored prognostic tools for this entity [26]. Secondly, the NCDB records overall survival (OS) but not cancer-specific survival or recurrence-free survival (RFS). While a high RS predicts a greater risk of distant recurrence, our use of OS may underestimate the assay’s true prognostic power for breast cancer-specific events, as it incorporates non-cancer deaths. Furthermore, the relatively low number of death events (n = 41), particularly within the high-risk subgroup receiving chemotherapy, limits the statistical power of our subgroup analyses. This low event rate precludes definitive conclusions about the absolute benefit of chemotherapy in high-risk IMPC patients and emphasizes the need for larger, collaborative studies to adequately power such analyses.

In summary, our study is the first to demonstrate in a large IMPC cohort that the Oncotype DX score stratifies overall survival, with high scores indicating markedly poorer prognosis. The data validate the predictive ability of the 21-gene assay in this distinct subtype, but also highlight that the conventional low vs. intermediate risk delineation may not carry the same weight in IMPC as in IDC-NST. These insights should be interpreted considering IMPC’s unique clinicopathologic behavior and the assay’s developmental context. Going forward, prospective research and possibly IMPC-specific molecular profiling are warranted to refine risk assessment in this disease. In the meantime, clinicians should continue to utilize Oncotype DX for IMPC as an aid in decision-making, recognizing its limitations but leveraging its ability to flag truly high-risk patients who stand to gain from intensified therapy to ultimately improve outcomes for this rare breast cancer subtype.

## Figures and Tables

**Table 1:** Baseline patient demographic and clinicopathologic characteristics of the study cohort, stratified by Oncotype DX Recurrence Score (RS) group (N = 1325).

Characteristics	Category	Total	≤11 N (%)	12–25 N (%)	>25 N (%)	Chi-Square *p*-Value
Age	<60	508	192 (37.8)	223 (43.9)	93 (18.31)	**0.0003**
	≥60	817	401 (49.08)	302 (36.96)	114 (13.95)	
Comorbidity score	0	1100	499 (45.36)	433 (39.36)	168 (15.27)	0.4171
	1	164	73 (44.51)	66 (40.24)	25 (15.24)	
	≥2	61	21 (34.43)	26 (42.62)	14 (22.95)	
Race	Non-White	221	88 (39.82)	91 (41.18)	42 (19)	0.1697
	White	1104	505 (45.74)	434 (39.31)	165 (14.95)	
Insurance	Medicare/Uninsured	634	303 (47.79)	246 (38.8)	85 (13.41)	**0.0381**
	Private	691	290 (41.97)	279 (40.38)	122 (17.66)	
Grade	Well differentiated	77	42 (54.55)	34 (44.16)	1 (1.3)	**<0.0001**
	Moderately differentiated	853	438 (51.35)	333 (39.04)	82 (9.61)	
	Poorly differentiated	374	106(28.34)	147 (39.3)	121 (32.35)	
	Undifferentiated	21	7 (33.33)	11 (52.38)	3 (14.29)	
Lumpectomy	No	400	157 (39.25)	178 (44.5)	65 (16.25)	**0.0239**
	Yes	925	436 (47.14)	347 (37.51)	142 (15.35)	
Radiation	No	442	185 (41.86)	185 (41.86)	72 (16.29)	0.3216
	Yes	883	408 (46.21)	340 (38.51)	135 (15.29)	
Chemotherapy	No	1057	578 (54.68)	434 (41.06)	45 (4.26)	**<0.0001**
	Yes	262	13 (4.96)	87 (33.21)	162 (61.83)	

Bolded values indicate variables with statistically significant associations by chi-square analysis.

**Table 2:** Five-year survival outcomes by Oncotype DX recurrence score in invasive micropapillary carcinoma (N = 1325). High-risk scores (>25) showed significantly higher mortality (6.2%) versus low/intermediate groups (2.4–2.5%, *p* = 0.0157).

Oncotype	Alive at 5 Years	Dead at 5 Years	Total
≤11	578 (97.5)	15 (2.5)	593
12–25	512 (97.5)	13 (2.4)	525
>25	194 (93.7)	13 (6.2)	207

*p*-value = 0.0157.

**Table 3:** Univariable and Multivariable Cox proportional hazards model for 5-year survival risk.

Variables		Univariable Hazard Ratio (95% CI)	Multivariable Hazard Ratio (95% CI)
Oncotype	≤11	**0.41 (0.20, 0.87)**	**0.31 (0.11, 0.88)**
	12–25	**0.38 (0.18, 0.83)**	**0.32** **(0.12, 0.86)**
	>25	ref	ref
Age	<60	**0.43 (0.20, 0.90)**	0.48 (0.21, 1.09)
	≥60	ref	ref
Race	Non-White	0.58 (0.21–1.63)	0.56 (0.20, 1.58)
	White	ref	ref
Number of comorbidities	0 vs. ≥2	**0.21 (0.1, 0.52)**	**0.25 (0.10, 0.61)**
	1 vs. ≥2	0.43 (0.15, 1.25)	0.41 (0.14, 1.19)
	≥2	ref	ref
Grade	Moderate vs. Well	0.88 (0.27, 2.9)	0.84 (0.25, 2.82)
	Poor vs. Well	0.62 (0.17, 2.29)	0.44 (0.11, 1.76)
	Undifferentiated vs. Well	1.14 (0.12, 10.9)	0.72 (0.07, 7.30)
	Well	ref	ref
Insurance	Medicare/Uninsured	**1.98 (1.05, 3.73)**	1.34 (0.66, 2.73)
	Commercial	ref	ref
Lumpectomy	No	**2.00 (1.08, 3.69)**	0.96 (0.37, 2.45)
	Yes	ref	ref
Chemotherapy	No	0.71 (0.34, 1.41)	1.12 (0.41, 3.00)
	Yes	ref	ref
Radiation	No	**2.61 (1.41, 4.84)**	**2.68 (1.05, 6.86)**
	Yes	ref	ref

Interpretation: Even after adjusting for covariates, Oncotype remains a significant predictor of 5-year survival, with lower oncotype associated with reduced risk of mortality. Bolded values indicate variables with statistically significant associations by chi-square analysis.

## Data Availability

The datasets analyzed during the current study are stored in an institutional repository and will be made available by the corresponding author on reasonable request.
